# Fishery-Independent Data Reveal Negative Effect of Human Population Density on Caribbean Predatory Fish Communities

**DOI:** 10.1371/journal.pone.0005333

**Published:** 2009-05-06

**Authors:** Christopher D. Stallings

**Affiliations:** Department of Zoology, Oregon State University, Corvallis, Oregon, United States of America; University of North Carolina at Chapel Hill, United States of America

## Abstract

**Background:**

Understanding the current status of predatory fish communities, and the effects fishing has on them, is vitally important information for management. However, data are often insufficient at region-wide scales to assess the effects of extraction in coral reef ecosystems of developing nations.

**Methodology/Principal Findings:**

Here, I overcome this difficulty by using a publicly accessible, fisheries-independent database to provide a broad scale, comprehensive analysis of human impacts on predatory reef fish communities across the greater Caribbean region. Specifically, this study analyzed presence and diversity of predatory reef fishes over a gradient of human population density. Across the region, as human population density increases, presence of large-bodied fishes declines, and fish communities become dominated by a few smaller-bodied species.

**Conclusions/Significance:**

Complete disappearance of several large-bodied fishes indicates ecological and local extinctions have occurred in some densely populated areas. These findings fill a fundamentally important gap in our knowledge of the ecosystem effects of artisanal fisheries in developing nations, and provide support for multiple approaches to data collection where they are commonly unavailable.

## Introduction

It is well documented that humans have greatly altered predatory fish communities worldwide, especially through industrialized commercial and recreational fisheries [1]–[8]. These studies have based their conclusions on extensive databases of fisheries-dependent data (i.e., landings statistics), primarily from developed nations. However, fisheries statistics are commonly unavailable in developing nations where artisanal (subsistence or small-scale commercial) fisheries exist [9]–[11]. Despite the problem of insufficient data, it remains imperative to assess region-wide effects of extraction on predatory fish populations and to indicate whether indirect effects of human activities exist in the communities to which they belong (e.g., dominance shifts) in order to implement management and conservation strategies geared towards ecosystem-based approaches [12].

Artisanal fisheries supply food for millions of people in developing nations, and are the primary source of resource exploitation on coral reef systems [13]. Fishing on Caribbean reefs occurred long before the arrival of European settlers, but has returned increasingly diminished yields over the last 200 years as human populations have escalated in the region [14]–[16]. Similar to industrial and recreational counterparts in developed nations, artisanal fishing tends to target large-bodied, top trophic-level fishes, so greater numbers of fishermen per unit area should result in increased removal of larger species [17]–[20]. Indeed, populations of large-bodied fishes have become notoriously impoverished at some Caribbean locations with high densities of human populations (e.g., Jamaica) [21], [22]. However, because fisheries data are generally unavailable or incomplete across the Caribbean, researchers have relied on either survey data from studies conducted on relatively small spatial scales or anecdotal and historical information. Therefore, the prevalence of these patterns and their potential indirect effects across the region remain unknown.

**Figure 1:**
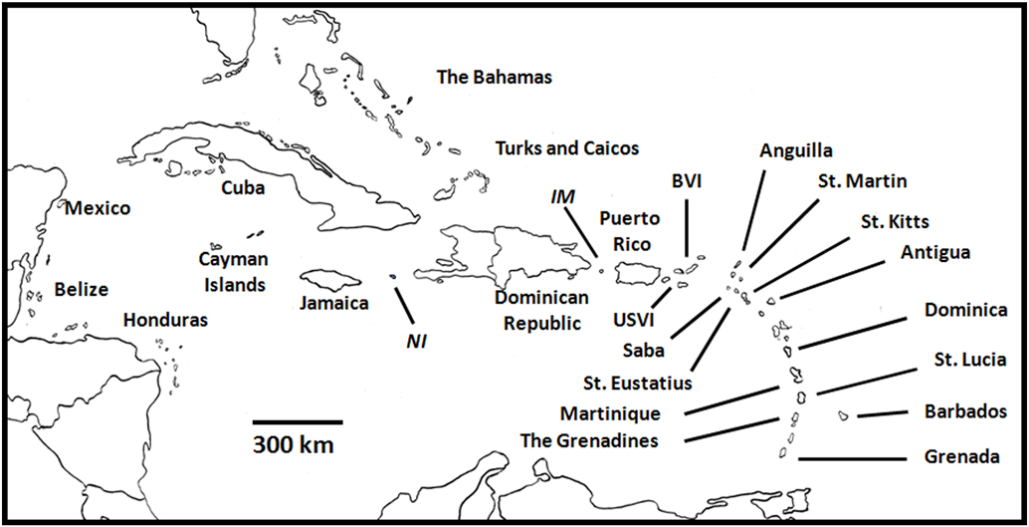
Map of Caribbean locations from which predator presence data were gathered. The data were from all locations in which at least 10 volunteer diver surveys were conducted between 1994 and 2008. The locations of the two uninhabited islands are italicized: *IM* (Isla de Mona); *NI* (Navassa Island).

To address these issues on a larger scale, I used a publicly accessible, fisheries-independent database [23] to provide the first broad scale, quantitative analysis of the structure of predatory reef-fish communities across the greater Caribbean region (Fig. 1). The database consisted of over 38,000 presence/absence surveys conducted across 22 insular and continental nations (Table 1) by citizen scientists (i.e., trained volunteer SCUBA divers), a technique that has been used extensively by terrestrial ecologists (e.g., Breeding Bird Survey), but largely ignored by their marine colleagues. These community efforts can cover large geographic scales and produce sample sizes several order of magnitude greater than traditional efforts by either individual or small teams of scientists [24], effectively filling data gaps where fisheries-dependent data are currently unavailable. I also examined potential mechanisms, including factors that are both independent of and related to anthropogenic influences (Table 2), that may have affected the structure of these fish communities.

**Table 1:** Twenty-two nations from which REEF survey data were collected, including information of human population densities and sample sizes.

Country/region	HPD	Code	Survey locations	Total surveys
Belize	12	BZ	7	2304
Bahamas	21	BA	15	9457
Turks and Caicos	47	TC	10	3136
Mexican Caribbean	53	MC	5	5057
Honduras	62	HD	4	2124
Cuba	102	CU	3	567
Leeward Islands		LI	8	1819
--- Anguilla	129			13
--- Netherlands Antilles*	131			600
--- St. Kitts	149			285
--- Antigua	155			27
--- Dominica	91			894
British Virgin Islands	147	BV	3	2196
Cayman Islands	168	CI	4	4499
Dominican Republic	183	DR	4	515
Jamaica	248	JA	5	384
US Virgin Islands	308	UV	3	2347
Windward Islands		WI	8	2635
--- Martinique	359			163
--- St. Lucia	269			181
--- St. Vincent & The Grenadines†	302			1929
--- Barbados	647			173
--- Grenada	260			189
Puerto Rico	430	PR	7	1076
				**TOTAL = 38116**

* Netherlands Antilles (St Martin, Saba, St Eustatius).

† St Vincent & Grenadines (includes Bequia & Mustique).

**Table 2:** Pearson's correlations (*r*) between explanatory variables and the axes from the NMS ordination.

Variable	Axis 1	Axis 2
HPD (people/land km^2^)	0.72	−0.01
HPReef (people/reef km^2^)	0.09	0.05
GDP (PPP/capita)	−0.18	−0.08
Tourist (mean/year)	−0.23	0.11
Latitude	−0.64	−0.07

## Results

**Figure 2:**
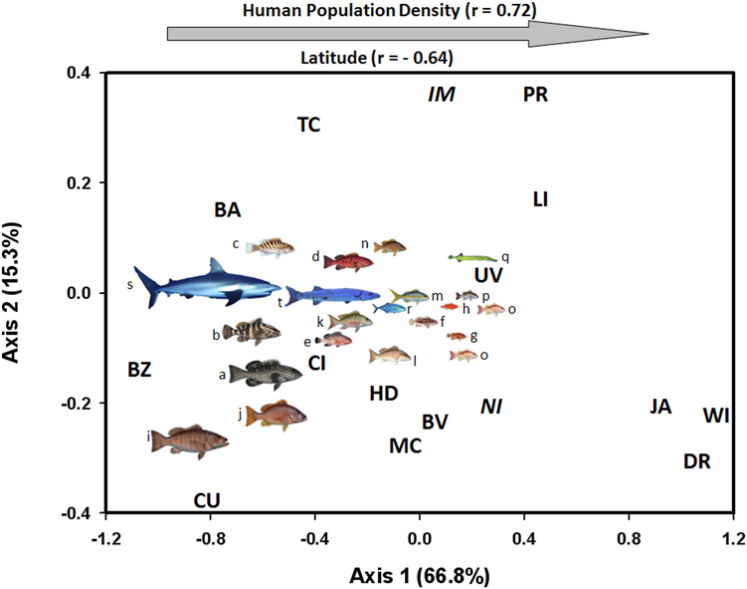
NMS ordination of regions in predatory fish space (20 taxa). Regional centroids are displayed: BA (Bahamas); TC (Turks and Caicos); CU (Cuba); CI (Cayman Islands); JA (Jamaica); MC (Mexican Caribbean); BZ (Belize); HD (Honduras); DR (Dominican Republic), PR (Puerto Rico); UV (US Virgin Islands); BV (British Virgin Islands); LI (Leeward Islands); WI (Windward Islands). The axis 1 scores for the two uninhabited islands are italicized: *IM* (Isla de Mona); *NI* (Navassa Island). Along axis 1, latitude increases towards the left and human population density increases towards the right. Taxa locations are represented with coded fish displays: a (*Mycteroperca bonaci*); b (*Epinephelus striatus*); c (*M. tigris*); d (*M. venenosa*); e (*E. guttatus*); f (*E. adscensionis*); g (*Cephalopholis cruentata*); h (*C. fulva*); I (*Lutjanus cyanopterus*); j (*L. jocu*); k (*L. analis*); l (*L. griseus*); m (*Ocyurus chrysurus*); n (*L. apodus*); o (*L. synagris*); p (*L. mahogoni*); q (*Aulostomus maculatus*); r (*Caranx* spp.); s (*Carcharhinus* spp.); t (*Sphyraena barracuda*). Fish displays are scaled according to maximum attainable sizes of each taxa.

A non-metric multidimensional scaling (NMS) ordination of 20 predatory taxa converged on a stable, 2-dimensional solution (final stress = 16.53, final instability = 0.00048, iterations = 74) (Fig. 2). The first axis accounted for the majority of variation in the NMS (*r^2^* = 0.67), was strongly correlated with human population density (*r* = 0.72) and slightly less so with latitude (*r* = −0.64; Table 2). The structure of the ordination was driven by strong associations of sharks (Carcharhinidae), jacks (Carangidae), and large species of groupers (Serranidae) and snappers (Lutjanidae) with regions of low human population density (high latitude). The pattern was also driven by moderate associations of trumpetfish (Aulostomidae) and smaller species of groupers and snappers with regions of high human population density (low latitude; Fig. 2). The second axis accounted for less variation (*r^2^* = 0.15) and was driven by regional differences in which particular taxa of large or small predators predominated.

Because human population density and latitude were the primary factors related to the structure of the NMS ordination along the first axis, a multiple regression was used to investigate their independent effects. Although human population densities tend to decrease towards higher latitudes in the Caribbean region (*r* = −0.57), collinearity was low (variance inflation factor = 1.469); therefore the analysis was deemed robust. Both human population density (p<0.00001) and latitude (p = 0.0121) were related to the NMS scores after accounting for the effects of each. However, analysis of the standardized regression coefficients (1 standard deviation) revealed stronger evidence for a significant effect of human population density on NMS scores compared to latitude (i.e., lower p-values), and that the effect of the former (coef_standardized_ = 0.4583) was over twice as strong as the latter (coef_standardized_ = −0.2126).

Mean and median sighting frequencies of predators decreased 2.2–4.0% (*r^2^* = 0.19, *p*<0.0001) and 4.1–7.1% (*r^2^* = 0.37, *p*<0.0001), respectively, per incremental increase of 100 humans per km^2^. The predator communities exhibited lower richness (*r^2^* = 0.20, *p*<0.0001) and Simpson's diversity (*r^2^* = 0.41, *p*<0.0001) with increasing density of humans. At the taxon level, 15 of the 20 predators included in the analyses were sighted less frequently with increasing human population density (Table 3). The remaining five predatory taxa were sighted either evenly or at increasing frequencies with increasing human population density, and included the smallest species of grouper (graysby, *Cephalopholis cruentata* and coney, *C. fulva*) and snapper (mahogany snapper, *Lutjanus mahogoni* and lane snapper, *L. synagris*), as well as the relatively unfished trumpetfish (*Aulostomus maculatus*).

**Table 3:** Regression statistics of predatory reef-fish presence across a gradient of human population density.

Family	Taxa	Common name	TL_max_ (cm)	Intercept	SE	Coef	SE	*t-Value*	*p-Value* C
Aulostomidae	*Aulostomus maculatus*	trumpetfish	100	0.4827	0.0306	0.0005	0.0002	3.089	0.0027*
Carangidae	*Caranx* spp.	jacks^a^	69^b^	0.7690	0.0242	−0.0003	0.0001	−2.374	0.0199
Carcharhinidae	*Carcharhinus* spp.	requiem sharks^a^	300^b^	0.0887	0.0142	−0.0002	0.0001	−4.152	0.0001*
Lutjanidae	*Lutjanus cyanopterus*	cubera snapper^a^	160	0.0672	0.0095	−0.0002	0.0000	−5.572	<0.0001*
	*L. jocu*	dog snapper^a^	128	0.0975	0.0142	−0.0001	0.0001	−2.131	0.0361
	*L. analis*	mutton snapper^a^	94	0.1659	0.0198	−0.0002	0.0001	−1.770	0.0805
	*L. griseus*	gray snapper	89	0.1551	0.0165	−0.0002	0.0001	−2.568	0.0120
	*Ocyurus chrysurus*	yellowtail snapper	86	0.7602	0.0272	−0.0004	0.0001	−2.980	0.0038*
	*L. apodus*	schoolmaster	67	0.6091	0.0338	−0.0006	0.0002	−3.606	0.0005*
	*L. synagris*	lane snapper	60	0.0509	0.0126	0.0002	0.0001	3.015	0.0034*
	*L. mahogoni*	mahogany snapper	48	0.3445	0.0304	0.0003	0.0002	1.992	0.0497
Serranidae	*Mycteroperca bonaci*	black grouper^a^	148	0.1810	0.0190	−0.0006	0.0001	−6.858	<0.0001*
	*Epinephelus striatus*	Nassau grouper^a^	122	0.4607	0.0321	−0.0013	0.0002	−9.206	<0.0001*
	*M. tigris*	tiger grouper^a^	101	0.3112	0.0251	−0.0009	0.0001	−7.882	<0.0001*
	*M. venenosa*	yellowfin grouper^a^	100	0.0358	0.0042	−0.0001	0.0000	−4.753	<0.0001*
	*E. guttatus*	red hind^a^	76	0.0090	0.0015	−0.0001	0.0000	−3.778	0.0003*
	*E. adscensionis*	rock hind^a^	61	0.0873	0.0138	−0.0001	0.0001	−1.366	0.1756
	*Cephalopholis cruentata*	graysby	43	0.4705	0.0305	0.0004	0.0002	2.510	0.0140
	*C. fulva*	coney	41	0.4632	0.0385	0.0004	0.0002	1.873	0.0646
Sphyraenidae	*Sphyraena barracuda*	barracuda	200	0.4616	0.0278	−0.0006	0.0001	−4.447	<0.0001*

a Regression coefficient and intercept values computed from untransformed data; test statistics computed from arcsine(ˆ0.5) transformed data (Zar 1999).

b Size data for sharks and jacks are from Caribbean reef shark (*Carcharhinus perezii*) and bar jack (*Caranx ruber*), respectively, which were the most common family representatives.

C Significant test after correction for multiple comparisons using sequential Bonferroni noted (*).

*Note:* Barbados was removed from the regressions since its high HPD (642people/km^2^) was approximately 50% greater than the second highest nation (i.e., outlier), and therefore quantitatively exaggerated the effect of HPD; trends were qualitatively unaffected.

**Figure 3:**
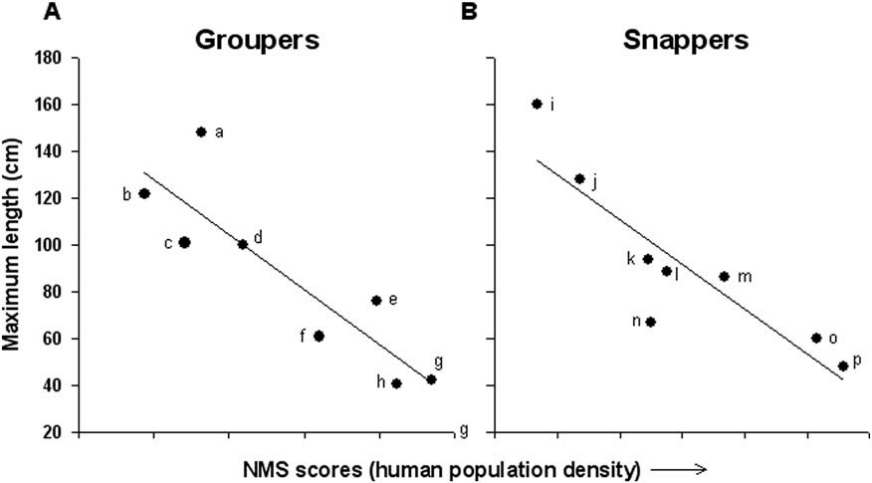
Maximum lengths of serranids and lutjanids as a function of human population density. Taxon codes are in caption to Figure 2. Regression statistics (*n* = 8 species each): (A) serranid maximum published lengths (*r^2^* = 0.78, *p* = 0.004); (B) lutjanid maximum published lengths (*r^2^* = 0.78, *p* = 0.003). NMS scores are from the axis that accounted for the most variation in the data. Axis variation explained and correlation with human population density: (A) serranid ordination (axis *r^2^* = 0.55, *r* with axis = 0.75); (B) lutjanid ordination (axis *r^2^* = 0.46, *r* with axis = 0.50).

NMS ordinations within both the grouper (final stress = 11.18, final instability = 0.00045, iterations = 59) and snapper (final stress = 11.21, final instability = 0.00045, iterations = 59) families each converged on stable, 3-dimensional solutions. The first axes of both ordinations accounted for the majority of variation (grouper *r^2^* = 0.55; snapper *r^2^* = 0.59) and were strongly correlated with human population density (grouper *r* = 0.75; snapper *r* = 0.57). Linear regressions within both families indicated strong decreases in maximum sizes of the species associations with regions along an index from low to high human population densities (Fig. 3).

## Discussion

The analyses presented here suggest human population density is strongly, negatively related to both richness and total presence (a surrogate of abundance) of predatory reef fishes in the Caribbean. Large predatory species were rare or absent in locations of high human population density, where smaller predators have become dominant, indicating the potential of indirect effects through competitive release. Although correlatives with both human activities and latitude may have had an influence on the structure of predatory communities, fishing was likely the most important mechanism driving the documented patterns.

Human population density and latitude were both correlated with the ordination of all taxa, but close examination of the data allow the relative effects of each predictor to be disentangled. In addition to compelling results from multiple regression analyses (see Results), further evidence reinforces that human population density was the dominant signal. First, although all taxa included in the analysis are naturally distributed across all locations in the study region, most fishes, particularly the larger-bodied ones, were rare or completely absent in surveys conducted in areas of high human population density. These patterns were evident in both the compressed, multivariate space (i.e., all large-bodied predators grouped on the left/negative side of axis 1, Fig. 2) and in the presence data of individual taxa (Table 3). In addition, historical data further illustrate that large groupers, snappers, and sharks were once abundant throughout the Caribbean, including reefs located in the Greater and Lesser Antilles where several of the species examined here are now ecologically or locally extinct [15], [25], [26].

Second, comparisons between inhabited and uninhabited islands within otherwise densely populated regions highlight potential human induced effects [27], [28]. For example, Isla de Mona and Navassa Island are uninhabited, relatively isolated nature reserves near the densely populated islands of Puerto Rico and Jamaica, respectively (Fig. 1). Although both islands have historically been fished and have experienced other anthropogenic effects, the intensity of such effects on these relatively remote locations is likely lower compared to nearby inhabited islands. Indeed, the similarities between the predator communities at these locales and other locations of low human density can be detected in both the ordinated space (i.e., italicized locations *IM* and *NI* further to the left on axis 1 than centroids of neighboring PR and JA, respectively, Fig. 2) and the presence/absence data for each taxon. Sighting frequencies of large-bodied predators, such as sharks, jacks, barracuda, and large groupers and snappers, were two to three times higher on reefs adjacent to the uninhabited islands relative to nearby inhabited ones (Text S1, Table S1). The more extensive presence of these predators within regions where they are otherwise rare or completely absent indicates that anthropogenic effects, not latitudinal gradients, limit the presence of these large-bodied fishes.

The relationship between human population density and ecological communities has been investigated far more extensively in terrestrial systems than marine ones [29]. However, several recent studies from the Line Islands [20], [30], [31] and the Hawaiian Islands [27], [32] have found higher abundances and biomass of large predatory fishes in locations of low human population densities compared to those that are densely populated. Similar results were found in the current study, with large predators becoming increasingly rare or locally extinct with increasing human population densities. Human activities can negatively affect populations and communities of coral reef fishes directly through harvesting and indirectly through habitat loss [32]. Worldwide degradation of coral reefs has been well documented [33]–[35], and although the effects of global climate change (and associated effects of bleaching, acidification, and disease) are thought to be the major drivers, local effects related to human population density (e.g., destructive fishing, pollution) exacerbate the destruction to coral habitats [36]–[41]. Decreased coral cover can result in declines to the abundance, biomass, and diversity of coral reef fishes [42]–[46], but most evidence is for small fishes occupying lower trophic levels, while that for predatory fishes is less clear. For example, Wormald [47] found varying relationships (positive and negative) of coral volume on two snappers (schoolmaster and lane snapper, respectively) while Graham et al. [43] was unable to detect a relationship between coral loss and fishes larger than 20 cm. Using meta-analysis, Paddack et al. [45] suggested declines in Caribbean fishes from several trophic groups were due to loss of coral, but were unable to detect a significant effect of habitat degradation on piscivores. Separating the effects of habitat loss from those of fishing have proven difficult since they commonly co-occur [48], but Williams et al. [32] was able to do so and concluded fishing to be the dominant factor affecting Hawaiian fish communities. The effects of fishing generally precede other stressors [49] and typically have the strongest human induced consequences on predatory marine fishes [18], [40], [50]. Although multiple and interactive local effects related to increasing human population density cannot be ignored, fishing is the most parsimonious mechanism driving the loss of predatory fishes in the Caribbean.

Artisanal fishing is the predominant source of resource extraction on coral reefs in the Caribbean [51]. Although commonly considered to be relatively benign compared to industrialized fisheries, increasing evidence from around the world suggests otherwise. Even at relatively low fishing intensities, artisanal fishing has been shown to strongly reduce populations and biomass of targeted species on coral reefs in the Indo-Pacific [52]–[54], eastern Pacific [55], and the Caribbean [18]. Fishermen tend to target and directly reduce populations of large-bodied fishes that are typically longer lived, mature more slowly than smaller ones, and often form spawning aggregations, all of which increase their vulnerability to overfishing [56]–[60]. Fishing can also have indirect effects on predatory fish communities. For example, removal of large-bodied predators may have allowed smaller ones to increase in abundance due to release from competition or predation [30], [61], [62]. Indeed, the relatively unfished trumpetfish, and the two smallest species of both grouper (i.e., graysby and coney) and snapper (i.e., lane and mahogany snappers) were found to increase in presence with decreasing presence of large predators (Table 3, Figs. 2 and 3). Although the temporal trends were not significant, it is notable that only graysby, lane snapper, and mahogany snapper exhibited increasing presence across the 15-year period of surveys (Text S2, Tables S2 and S3).

Latitude was the second strongest correlative with the structure of predatory fish communities (Table 2). Most studies that have addressed latitudinal patterns of fish communities in the western Atlantic have done so across biogeographic provinces [63], [64], while few have been confined to the greater Caribbean and none have focused solely on predators in the region. Temperature and productivity can each vary greatly over large spatial scales and both have been linked to species richness gradients in the Atlantic [65] and Indo-Pacific [66]. However, neither annual temperature [67] nor productivity [68] varies greatly across the relatively warm, oligotrophic waters of the current study; their roles in affecting the structure of reef fish communities in the Caribbean, including that of the predatory fishes examined here, has therefore remained elusive. In a study that included various habitats including coral reefs, Bouchon-Navaro et al. [69] found latitude to explain a small but significant amount of the variance (8.4%) on the structure of fish assemblages across the Antilles, with increasing species richness towards lower latitudes. The authors suggested the patterns may have been attributable to the types and area of available habitat, but also acknowledged that it is difficult to attribute mechanism to latitudinal gradients of fishes in the Caribbean given our current knowledge. Following island biogeography theory [70], Sandin et al. [71] found fish richness on Caribbean reefs from insular nations to increase with both island area and decreasing isolation. Although distance between islands in the Caribbean tends to increase towards lower latitudes (*r* = between 0.40 and 0.65, depending on metric of isolation), richness was not correlated with latitude *per se* (*r* = −0.08; S.A. Sandin, unpublished data). Therefore the mechanisms behind the latitude signal in the current study are not very clear, but may have been due to a combination of gradients in both isolation and area of reefs confounded by the effects of human population density in a general north-south orientation.

The remaining three factors explained far less variance in the structure of predatory fish communities. The lack of a strong signal from the tourism data (i.e., the number of visitors) was somewhat surprising, since increased number of tourists should theoretically have had effects similar to those of increased number of residents. However, a recent study from the Bahamas indicated that residents account for the vast majority of seafood consumed (88%) compared to tourists, with the former preferring fishes (especially grouper and snapper) and the latter preferring conch and lobster (unpublished data, L. Talaue-McManus). Chronic demand for seafood from residents (particularly fishes) may supersede the effects from visitors.

Predicting the ecological consequences of changes to the structure of predator communities is difficult [72], [73]. Different sized predatory fishes may perform various functional roles and can have drastically different effects on the diversity and abundance of prey species [74], [75]. Furthermore, loss of functional roles can lead to decreased ecological stability [76] and ecosystems can become both less resilient to catastrophic phenomena such as cyclones [39] and less resistant to invasions by exotic species [77]. The recent invasion of Indo-Pacific lionfishes (*Pterois volitans* and *P. miles*) in the Caribbean may have been facilitated by overfishing large predators capable controlling their rapid spread and population explosion [78] and is alarming considering the strong predatory effects lionfish can have on native fishes [79]. Management of human impacts on entire functional groups may therefore be more important than targeting specific taxa [80], but tests of functional redundancy among predatory marine fishes is sorely needed [81]. In addition, incorporating the effects of environmental variation [82], multiple human stressors [83], and linkages in interaction webs [84], [85] with socioeconomic factors that lead to overfishing [86] may improve management and conservation in coral reef systems.

On a global scale, 37% of human populations are within 100 km of a coastline [87]. As human populations continue to increase, the associated negative effects on coastal ecosystems are not likely to be easily resolved. Continued efforts at broad spatial scales are necessary to better understand individual and interactive effects of anthropogenic activities on marine ecosystems [19], [39], [88], [89]. If we are to overcome the challenges of collecting data in developing nations and on a region-wide scale, these studies will require multiple disciplinary approaches [90] including publicly available survey data collected by citizen scientists and other community volunteers.

## Materials and Methods

### Survey Data

Predator presence/absence data from locations across the greater Caribbean region (Fig. 1) were queried using the Reef Environmental Education Foundation's (REEF) online database (World Wide Web electronic publication; www.reef.org, date of download: 20 August 2008). The data included coral reef habitats located in 22 continental and insular nations and consisted of 38,116 surveys conducted between 1994 and 2008 (Text S3, Table S4). Within each of the 22 nations, I chose survey locations with a minimum of 10 surveys (Table 1; 86 total locations). The data were collected by trained volunteer SCUBA divers using the Roving Diver Technique (RDT) where divers swim freely around a survey site and record all species that can be positively identified [91]. The RDT was specifically designed for volunteer data and is effective at rapid assessment of both fish distribution and abundance [92].

The analysis included all predators (trophic level≥4) [93] that met two fundamental criteria: 1) previously documented natural distributions for each of the 22 nations [93]–[96], and 2) only data for conspicuous species because the data were collected by volunteer divers. Although cryptic species (e.g., moray eels, Muraenidae; lizardfishes, Synodontidae) were recorded by the divers, the accuracy of the RDT at estimating their presence was unclear, so those data were not included. Twenty taxa of predatory fishes met the above criteria and included eight species of grouper (Family Serranidae), eight species of snapper (Lutjanidae), one species each of trumpetfish (Aulostomidae) and barracuda (Sphyraenidae), and both jacks (Carangidae) and requiem sharks (Carcharhinidae) summarized at the family levels (Table 3). The 20 taxa ranged in maximum attainable total lengths from 40 cm to over 300 cm. The average depth of each survey was recorded by REEF participants in 10 feet (3.05 meter) increments. Across all surveys included in the analyses here, the majority of dives (82%) were made at depths between 10–30 m, with decreasing proportions made at shallower (<10 m; 12%) and deeper (30–45 m; 7%) depths. Importantly, all surveys were conducted within the natural depth ranges of the 20 predatory taxa [93]–[96].

### Data Analysis

The predator presence/absence data had extremely low Whitaker's beta diversity (β = 0.1) and low values of the coefficient of variation for both taxa (CV = 87.6) and sample locations (CV = 22.8); therefore data transformation was not required. To investigate spatial patterns in the data, a matrix of sample locations by taxa presence was ordinated using non-metric multidimensional scaling (NMS) [97], [98]. NMS can investigate potential drivers influencing the final structure of the ordination by examining correlations between the main dataset (i.e., predator presence) and variables in a second matrix. Therefore a second matrix was constructed that included four variables related to human influences as well as latitude to account for biogeographic patterns that may have naturally existed across the 22 nations (Table 2). The four variables related to human influences included: 1) the size of human populations corrected for land area (the standard measure of human population density) [99], 2) human population size corrected for reef area [99], [100], 3) per capita gross domestic product [101], and 4) average tourist arrivals per year [102].

The ordinations of sample locations in species space were presented graphically, with overlays of the environmental data from the second matrix. The presentation was simplified by displaying national centroids and by grouping nations from the Lesser Antilles into ‘Windward’ (i.e., Barbados, Grenada, Martinique, St. Lucia, St. Vincent and the Grenadines) and ‘Leeward’ (i.e., Anguilla, Antigua, Dominica, Netherlands Antilles, St. Kitts) islands. The resulting ordination displayed 14 regions across the greater Caribbean region. All NMS ordinations were conducted in PC-ORD 5.14 using the ‘Autopilot Mode’ with Sorensen distance measure and random starting configurations [103].

In addition to the ordination, linear regressions were conducted between human population densities and several metrics of the predator presence data per sample location: 1) mean and median presence across all taxa, 2) richness (*S*, the total number of species), and 3) Simpson's diversity (*D* = 1−Σ (p_i_^2^).

Groupers and snappers are among the most speciose families of predatory reef fishes in the Caribbean, with a range of maximum total lengths for the species included here from <0.5 m to >1.5 m. Therefore, additional NMS ordinations were conducted on both families to investigate their within family associations with the survey locations relative to the maximum sizes of each species. The first axes of both ordinations were strongly correlated with human population densities. The NMS scores therefore served as an index of human population density in multivariate space for both ordinations. The relationship between how sizes of the associated species changed across the index of human population densities was analyzed using linear regression of the NMS scores versus the maximum attainable lengths of each species.

## Supporting Information

Text S1 Comparisons between uninhabited and densely populated islands.
(0.03 MB DOC)

Text S2 Temporal trends in predator presence.
(0.03 MB DOC)

Text S3 Comparisons between different levels of REEF surveyor experience.
(0.04 MB DOC)

Table S1 Comparisons of average sighting frequencies between both Jamaica-Navassa and Puerto Rico-Mona island pairs.
(0.05 MB DOC)

Table S2 Regression statistics of the presence of predatory reef fishes across time (1994–2008) by human population density interaction.
(0.05 MB DOC)

Table S3 Regression statistics of the presence of predatory reef fishes across time (1994–2008).
(0.05 MB DOC)

Table S4 Tests of whether sighting frequency differed between novice and expert surveyors.
(0.05 MB DOC)

## References

[1] Christensen V, Guenette S, Heymans JJ, Walters CJ, Watson R (2003). Hundred-year decline of North Atlantic predatory fishes.. Fish and Fisheries.

[2] Coleman FC, Figueira WF, Ueland JS, Crowder LB (2004). The impact of United States recreational fisheries on marine fish populations.. Science.

[3] Essington TE, Beaudreau AH, Wiedenmann J (2006). Fishing through marine food webs.. Proceedings of the National Academy of Sciences of the United States of America.

[4] Myers RA, Worm B (2003). Rapid worldwide depletion of predatory fish communities.. Nature.

[5] Myers RA, Worm B (2005). Extinction, survival or recovery of large predatory fishes.. Philosophical Transactions of the Royal Society B-Biological Sciences.

[6] Pauly D, Christensen V, Dalsgaard J, Froese R, Torres F (1998). Fishing down marine food webs.. Science.

[7] Worm B, Barbier EB, Beaumont N, Duffy JE, Folke C (2006). Impacts of biodiversity loss on ocean ecosystem services.. Science.

[8] Worm B, Sandow M, Oschlies A, Lotze HK, Myers RA (2005). Global patterns of predator diversity in the open oceans.. Science.

[9] Polunin NVC, Roberts CM, Pauly D, Polunin NVC, Roberts CM (1996). Developments in tropical reef fisheries science and management.. Reef Fisheries.

[10] Russ GR, Sale PF (1991). Coral reef fisheries: effects and yields.. The Ecology of Fishes on Coral Reefs.

[11] Sadovy Y, Domeier M (2005). Are aggregation-fisheries sustainable? Reef fish fisheries as a case study.. Coral Reefs.

[12] Francis RC, Hixon MA, Clarke ME, Murawski SA, Ralston S (2007). Fisheries management - Ten commandments for ecosystem-based fisheries scientists.. Fisheries.

[13] Munro JL, Polunin NVC, Roberts CM (1996). The scope of tropical reef fisheries and their management.. Reef Fisheries.

[14] Jackson JBC (1997). Reefs since Columbus.. Coral Reefs.

[15] Jackson JBC (2001). What was natural in the coastal oceans?. Proceedings of the National Academy of Sciences of the United States of America.

[16] Wing SR, Wing ES (2001). Prehistoric fisheries in the Caribbean.. Coral Reefs.

[17] Abesamis RA, Russ GR (2005). Density-dependent spillover from a marine reserve: Long-term evidence.. Ecological Applications.

[18] Hawkins JP, Roberts CM (2004). Effects of artisanal fishing on Caribbean coral reefs.. Conservation Biology.

[19] Newton K, Cote IM, Pilling GM, Jennings S, Dulvy NK (2007). Current and future sustainability of island coral reef fisheries.. Current Biology.

[20] Stevenson C, Katz LS, Micheli F, Block B, Heiman KW (2007). High apex predator biomass on remote Pacific islands.. Coral Reefs.

[21] Hughes TP (1994). Catastrophes, phase shifts, and large-scale degradation of a Caribbean coral reef.. Science.

[22] Munro JL (1983). Coral reef fish and fisheries of the Caribbean Sea.. ICLARM Stud Rev.

[23] REEF (2008). Reef Environmental Education Foundation.. http://www.reef.org.

[24] Cohn JP (2008). Citizen science: Can volunteers do real research?. Bioscience.

[25] Dampier W (1729). A new voyage around the world.

[26] Levin PS, Grimes CB, Sale P (2002). Reef fish ecology and grouper conservation and management.. Coral Reef Fishes: Dynamics and Diversity in a Complex Ecosystem.

[27] Friedlander AM, DeMartini EE (2002). Contrasts in density, size, and biomass of reef fishes between the northwestern and the main Hawaiian islands: the effects of fishing down apex predators.. Marine Ecology Progress Series.

[28] Miller MW, Gerstner CL (2002). Reefs of an uninhabited Caribbean island: fishes, benthic habitat, and opportunities to discern reef fishery impact.. Biological Conservation.

[29] Luck GW (2007). A review of the relationships between human population density and biodiversity.. Biological Reviews.

[30] DeMartini EE, Friedlander AM, Sandin SA, Sala E (2008). Differences in fish-assemblage structure between fished and unfished atolls in the northern Line Islands, central Pacific.. Marine Ecology Progress Series.

[31] Sandin SA, Smith JE, DeMartini EE, Dinsdale EA, Donner SD (2008). Baselines and degradation of coral reefs in the northern Line Islands.. PLoS ONE.

[32] Williams ID, Walsh WJ, Schroeder RE, Friedlander AM, Richards BL (2008). Assessing the importance of fishing impacts on Hawaiian coral reef fish assemblages along regional-scale human population gradients.. Environmental Conservation.

[33] Bellwood DR, Hughes TP, Folke C, Nystrom M (2004). Confronting the coral reef crisis.. Nature.

[34] Bruno JF, Selig ER (2007). Regional decline of coral cover in the Indo-Pacific: timing, extent, and subregional comparisons.. PLoS ONE.

[35] Gardner TA, Cote IM, Gill JA, Grant A, Watkinson AR (2003). Long-term region-wide declines in Caribbean corals.. Science.

[36] Aronson RB, Bruno JF, Precht WF, Glynn PW, Harvell CD (2003). Causes of coral reef degradation.. Science.

[37] Carpenter KE, Abrar M, Aeby G, Aronson RB, Banks S (2008). One-third of reef-building corals face elevated extinction risk from climate change and local impacts.. Science.

[38] Hoegh-Guldberg O, Mumby PJ, Hooten AJ, Steneck RS, Greenfield P (2007). Coral reefs under rapid climate change and ocean acidification.. Science.

[39] Hughes TP, Baird AH, Bellwood DR, Card M, Connolly SR (2003). Climate change, human impacts, and the resilience of coral reefs.. Science.

[40] Mora C (2008). A clear human footprint in the coral reefs of the Caribbean.. Proceedings of the Royal Society B-Biological Sciences.

[41] Rogers C (2009). Coral bleaching and disease should not be underestimated as causes of Caribbean coral reef decline.. Proceedings of the Royal Society B-Biological Sciences.

[42] Cheal AJ, Wilson SK, Emslie MJ, Dolman AM, Sweatman H (2008). Responses of reef fish communities to coral declines on the Great Barrier Reef.. Marine Ecology Progress Series.

[43] Graham NAJ, McClanahan TR, MacNeil MA, Wilson SK, Polunin NVC (2008). Climate warming, marine protected areas and the ocean-scale integrity of coral reef ecosystems.. PLoS ONE.

[44] Jones GP, McCormick MI, Srinivasan M, Eagle JV (2004). Coral decline threatens fish biodiversity in marine reserves.. Proceedings of the National Academy of Sciences of the United States of America.

[45] Paddack MJ, Reynolds JD, Aguilar C, Appeldoorn RS, Beets J (in press). Recent region-wide declines in Caribbean reef fish abundance.. Current Biology.

[46] Syms C, Jones GP (2000). Disturbance, habitat structure, and the dynamics of a coral-reef fish community.. Ecology.

[47] Wormald CL (2007).

[48] Sadovy Y (2005). Trouble on the reef: the imperative for managing vulnerable and valuable fisheries.. Fish and Fisheries.

[49] Jackson JBC, Kirby MX, Berger WH, Bjorndal KA, Botsford LW (2001). Historical overfishing and the recent collapse of coastal ecosystems.. Science.

[50] Jenkins M (2003). Prospects for biodiversity.. Science.

[51] Breton Y, Brown DN, Haughton M, Ovares L, Breton Y, Brown DN, Davy B, Haughton M, Ovares L (2006). Social sciences and the diversity of Caribbean communities.. Coastal Resource Management in the Wider Caribbean: Resilience, Adaptation, and Community Diversity.

[52] Jennings S, Polunin NVC (1996). Effects of fishing effort and catch rate upon the structure and biomass of Fijian reef fish communities.. Journal of Applied Ecology.

[53] McClanahan TR, Hicks CC, Darling ES (2008). Malthusian overfishing and efforts to overcome it on Kenyan coral reefs.. Ecological Applications.

[54] Russ GR, Alcala AC (1996). Marine reserves: Rates and patterns of recovery and decline of large predatory fish.. Ecological Applications.

[55] Ruttenberg BI (2001). Effects of artisanal fishing on marine communities in the Galapagos Islands.. Conservation Biology.

[56] Huntsman GR, Potts J, Mays RW, Vaughan D, Musick JA (1999). Groupers (Serranidae, Epinephelinae): Endangered Apex Predators of Reef Communities;.

[57] Jennings S, Reynolds JD, Polunin NVC (1999). Predicting the vulnerability of tropical reef fishes to exploitation with phylogenies and life histories.. Conservation Biology.

[58] Levin PS, Grimes CB, Sale P (2002). Reef fish ecology and grouper conservation and management.. Coral Reef Fishes: Dynamics and Diversity in a Complex Ecosystem.

[59] Roberts CM (1997). Ecological advice for the global fisheries crisis.. Trends in Ecology & Evolution.

[60] Sala E, Ballesteros E, Starr RM (2001). Rapid decline of Nassau grouper spawning aggregations in Belize: Fishery management and conservation needs.. Fisheries.

[61] Fogarty MJ, Murawski SA (1998). Large-scale disturbance and the structure of marine system: Fishery impacts on Georges Bank.. Ecological Applications.

[62] Watson M, Ormond RFG (1994). Effect of an artisanal fishery on the fish and urchin populations of a Kenyan coral reef.. Marine Ecology Progress Series.

[63] Briggs JC (1974). Marine Zoogeography.

[64] Floeter SR, Rocha LA, Robertson DR, Joyeux JC, Smith-Vaniz WF (2008). Atlantic reef fish biogeography and evolution.. Journal of Biogeography.

[65] Macpherson E (2002). Large-scale species-richness gradients in the Atlantic Ocean.. Proceedings of the Royal Society of London Series B-Biological Sciences.

[66] Bellwood DR, Hughes TP, Connolly SR, Tanner J (2005). Environmental and geometric constraints on Indo-Pacific coral reef biodiversity.. Ecology Letters.

[67] Leichter JJ, Helmuth B, Fischer AM (2006). Variation beneath the surface: Quantifying complex thermal environments on coral reefs in the Caribbean, Bahamas and Florida.. Journal of Marine Research.

[68] Dandonneau Y, Deschamps PY, Nicolas JM, Loisel H, Blanchot J (2004). Seasonal and interannual variability of ocean color and composition of phytoplankton communities in the North Atlantic, equatorial Pacific and South Pacific.. Deep-Sea Research Part II.

[69] Bouchon-Navaro Y, Bouchon C, Louis M, Legendre P (2005). Biogeographic patterns of coastal fish assemblages in the West Indies.. Journal of Experimental Marine Biology and Ecology.

[70] MacArthur RH, Wilson EO (1967). The theory of island biogeography.

[71] Sandin SA, Vermeij MJA, Hurlbert AH (2008). Island biogeography of Caribbean coral reef fish.. Global Ecology and Biogeography.

[72] Bruno JF, Cardinale BJ (2008). Cascading effects of predator richness.. Frontiers In Ecology And The Environment.

[73] Heithaus MR, Frid A, Wirsing AJ, Worm B (2008). Predicting Ecological Consequences of Marine Top Predator Declines.. Trends in Ecology and Evolution.

[74] Hixon MA, Carr MH (1997). Synergistic predation, density dependence, and population regulation in marine fish.. Science.

[75] Stallings CD (2008). Indirect effects of an exploited predator on recruitment of coral-reef fishes.. Ecology.

[76] McCann KS (2000). The diversity-stability debate.. Nature.

[77] Elton CS (1958). The ecology of invasions by plants and animals.

[78] Whitfield PE, Hare JA, David AW, Harter SL, Munoz RC (2007). Abundance estimates of the Indo-Pacific lionfish Pterois volitans/miles complex in the Western North Atlantic.. Biological Invasions.

[79] Albins MA, Hixon MA (2008). Invasive Indo-Pacific lionfish Pterois volitans reduce recruitment of Atlantic coral-reef fishes.. Marine Ecology Progress Series.

[80] Hughes TP, Bellwood DR, Folke C, Steneck RS, Wilson J (2005). New paradigms for supporting the resilience of marine ecosystems.. Trends in Ecology & Evolution.

[81] Stallings CD (in press). Predatory identity and recruitment of coral-reef fishes: indirect effects of fishing.. Marine Ecology Progress Series.

[82] Pikitch EK, Santora C, Babcock EA, Bakun A, Bonfil R (2004). Ecosystem-based fishery management.. Science.

[83] McLeod KL, Lubchenco J, Palumbi SR, Rosenberg AA (2005). Scientific Consensus Statement on Marine Ecosystem-Based Management.. http://compassonline.org/?q=EBM.

[84] Crowder LB, Hazen EL, Avissar N, Bjorkland R, Latanich C (2008). The Impacts of Fisheries on Marine Ecosystems and the Transition to Ecosystem-Based Management.. Annual Review of Ecology Evolution and Systematics.

[85] Walters C, Christensen V, Pauly D (1997). Structuring dynamic models of exploited ecosystems from trophic mass-balance assessments.. Reviews in Fish Biology and Fisheries.

[86] Cinner JE, McClanahan TR, Daw TM, Graham NAJ, Maina J (2009). Linking social and ecological systems to sustain coral reef fisheries.. Current Biology.

[87] Cohen JE, Small C, Mellinger A, Gallup J, Sachs J (1997). Estimates of coastal populations.. Science.

[88] Mumby PJ, Dahlgren CP, Harborne AR, Kappel CV, Micheli F (2006). Fishing, trophic cascades, and the process of grazing on coral reefs.. Science.

[89] Pandolfi JM, Jackson JBC, Baron N, Bradbury RH, Guzman HM (2005). Are US coral reefs on the slippery slope to slime?. Science.

[90] Pinnegar JK, Engelhard GH (2008). The ‘shifting baseline’ phenomenon: a global perspective.. Reviews in Fish Biology and Fisheries.

[91] Schmitt EF, Sullivan KM (1996). Analysis of a volunteer method for collecting fish presence and abundance data in the Florida Keys.. Bulletin of Marine Science.

[92] Schmitt EF, Sluka RD, Sullivan-Sealey KM (2002). Evaluating the use of roving diver and transect surveys to assess the coral reef fish assemblages off southeastern Hispaniola.. Coral Reefs.

[93] Froese R, Pauly D (2005). http://www.fishbase.org.

[94] Allen GR (1985). FAO species catalogue. Snappers of the world.

[95] Heemstra PC, Randall JE (1993). FAO species catalogue. Vol. 16. Groupers of the world (family Serranidae, subfamily Epinephelinae).

[96] Humann P, DeLoach N (2002). Reef fish identification.

[97] Kruskal JB (1964). Nonmetric multidimensional scaling: a numerical method.. Psychometrika.

[98] Mather PM (1976). Computational methods of multivariate analysis in physical geography.

[99] United Nations (2005). World Populations Prospects Report.

[100] Spalding MD, Ravilous C, Green EP (2001). World atlas of coral reefs.

[101] Central Intelligence Agency (2008). The world factbook.

[102] Caribbean Tourism Organization (2008). Tourist stop over arrivals.

[103] McCune B, Mefford MJ (1999). PC-ORD. Multivariate Analysis of Ecological Data. 5.14 beta ed.

