# Supplementary material for: Fishery-Independent Data Reveal Negative Effect of Human Population Density on Caribbean Predatory Fish Communities
Source: PLoS One. 2009 May 6;4(5):e5333. doi: 10.1371/journal.pone.0005333 (PMC2672166; doi:10.1371/journal.pone.0005333)
Supplement: Text S1 — Comparisons between uninhabited and densely populated islands. (0.03 MB DOC) [file pone.0005333.s001.doc]

**On-line supplementary material**

Comparisons between Uninhabited and Densely Populated Islands

The comparisons between uninhabited Navassa and Mona Islands with densely populated Jamaica and Puerto Rico, respectively, did not include a formal analysis per se. Instead, the goal was to highlight the strong differences in both the multivariate and univariate data between unpopulated and populated islands at the same latitudes. These comparisons indicate the average sighting frequency of large, targeted predators (i.e., those <100cm) was 2.98 times higher on Navassa Island and 2.43 times higher on Mona Island, than on Jamaica and Puerto Rico, respectively (Table S1).
