# Supplementary material for: Fishery-Independent Data Reveal Negative Effect of Human Population Density on Caribbean Predatory Fish Communities
Source: PLoS One. 2009 May 6;4(5):e5333. doi: 10.1371/journal.pone.0005333 (PMC2672166; doi:10.1371/journal.pone.0005333)
Supplement: Text S2 — Temporal trends in predator presence. (0.03 MB DOC) [file pone.0005333.s002.doc]

**On-line supplementary material**

Temporal Trends in Predator Presence

The data were analyzed to determine whether temporal trends existed in the presence of individual taxa (i.e., sighting frequency). Sighting frequency for each taxa and human population density were both summarized at the three-year scale described above. A linear regression of sighting frequency against the main and interactive terms for time and human population density was conducted first to determine if changes were contingent upon human population density. No tests of the interaction term were statistically significant (Table S2). A second linear regression was conducted with the interaction and human population density terms removed from the model. After correction for multiple comparisons using sequential Bonferroni [S1] only sighting frequency of trumpetfish decreased significantly over time (Table S3). Although none of the other 19 taxa displayed significant changes, it is notable that with the exception of the second smallest grouper (i.e., graysby) and the two smallest snappers (i.e., lane and mahogany), all taxa exhibited declining trends across the region. It is possible that the inability to detect significant trends over the 15-year period for some predators found at low abundances was due to low statistical power [S2]. In addition, the Caribbean had already been intensively affected by human activities at the onset of the REEF survey program in 1994, and trends since that time would suffer from the problem of shifting baselines [S3,S4]. Regardless, no strong temporal signal was found across the overall dataset. Therefore sighting frequencies for each taxa at each of the 86 survey locations were summarized across the entire 15-year period for the main analyses.

Supporting Text References

S1. Holm S (1979) A simple sequential rejective multiple test procedure. Scandinavian Journal of Statistics 6: 65-70.

S2. Maxwell D, Jennings S (2005) Power of monitoring programmes to detect decline and recovery or rare and vulnerable fish. Journal of Applied Ecology 42: 25-37.

S3. Pauly D (1995) Anecdotes and the shifting baseline syndrome of fisheries. Trends in Ecology and Evolution 10: 430.

S4. Pinnegar JK, Engelhard GH (2008) The 'shifting baseline' phenomenon: a global perspective. Reviews in Fish Biology and Fisheries 18(1): 1-16.
