# Supplementary material for: Fishery-Independent Data Reveal Negative Effect of Human Population Density on Caribbean Predatory Fish Communities
Source: PLoS One. 2009 May 6;4(5):e5333. doi: 10.1371/journal.pone.0005333 (PMC2672166; doi:10.1371/journal.pone.0005333)
Supplement: Text S3 — Comparisons between different levels of REEF surveyor experience. (0.04 MB DOC) [file pone.0005333.s003.doc]

**On-line supplementary material**

Surveyor Experience

REEF participants are separated into two categories, “Novice” and “Expert”, based on a combination of surveyor experience (i.e., number of surveys completed) and examination scores from fish identification tests. Although non-expert surveyors can be extremely effective at accurate fish identification and data collection when compared to experts [S1-S3], it was important to determine whether the two REEF data types differed in order to validate use of the combined data versus only those collected by expert surveyors. Therefore, an Analysis of Covariance (ANCOVA) was conducted for each of the 20 predatory taxa across the Caribbean with experience level as the covariate and taxa presence as the response across both human population density and latitude (i.e., 40 separate ANCOVA’s were conducted). Because the focus of this study was on the relative structures of predatory fish communities, not absolute abundances, comparisons focused on the slopes of novice and expert data rather than the intercepts. Only locations with more than 10 surveys for each experience level were included in the analyses, which resulted in sixty-five locations with 25,181 and 12,531 surveys conducted by novice and expert surveyors, respectively.

The slopes did not differ between expert and novice data types at the alpha = 0.05 level for each of the 40 ANCOVA’s conducted (i.e., 20 HPD*Experience, 20 Latitude*Experience) (Table S4). These results indicate that sighting frequencies for each of the 20 species did not differ between novice and expert surveyors across the study region. In addition, significance levels were not corrected for multiple comparisons and therefore provide conservative justification for using the total (i.e., combined) data.

Supporting Text References

S1. Harvey E, Fletcher D, Shortis M (2001) A comparison of the precision and accuracy of estimates of reef-fish lengths determined visually by divers with estimates produced by a stereo-video system. Fishery Bulletin 99(1): 63-71.

S2. Harvey E, Fletcher D, Shortis M (2001) Improving the statistical power of length estimates of reef fish: a comparison of estimates determined visually by divers with estimates produced by a stereo-video system. Fishery Bulletin 99(1): 72-80.

S3. Pattengill-Semmens CV, Semmens BX (1998) An Analysis of Fish Survey Data Generated by Nonexpert Volunteers in the Flower Garden Banks National Marine Sanctuary. Gulf of Mexico Science 16(2): 196-207.

S4. Central Intelligence Agency (annual reports: 1994-2008) The world factbook. Washington, DC: Central Intelligence Agency.
