# Supplementary material for: Fishery-Independent Data Reveal Negative Effect of Human Population Density on Caribbean Predatory Fish Communities
Source: PLoS One. 2009 May 6;4(5):e5333. doi: 10.1371/journal.pone.0005333 (PMC2672166; doi:10.1371/journal.pone.0005333)
Supplement: Table S1 — Comparisons of average sighting frequencies between both Jamaica-Navassa and Puerto Rico-Mona island pairs. (0.05 MB DOC) [file pone.0005333.s004.doc]

**On-line supplementary material**

Table S1. Comparisons of average sighting frequencies (%) between both Jamaica-Navassa and Puerto Rico-Mona island pairs.a

| Family | Taxa | Common name | TL_max_ (cm) |  | Jamaica | Navassa |  | Puerto Rico | Mona |
| --- | --- | --- | --- | --- | --- | --- | --- | --- | --- |
| Aulostomidae | *Aulostomus maculatus* | trumpetfish | 100 |  | 61.35 | 20.00 |  | 51.82 | 41.30 |
| Carangidae | *Caranx* spp. | jacks | 69^b^ |  | 61.93 | 53.30 |  | 74.70 | 91.30 |
| Carcharhinidae | *Carcharhinus* spp. | requiem sharks | 300^b^ |  | 1.97 | 0.00 |  | 1.53 | 1.10 |
| Lutjanidae | *Lutjanus cyanopterus* | cubera snapper | 160 |  | 0.00 | 0.00 |  | 0.32 | 0.00 |
|  | *L. jocu* | dog snapper | 128 |  | 2.77 | 6.70 |  | 5.30 | 3.30 |
|  | *L. analis* | mutton snapper | 94 |  | 23.10 | 6.70 |  | 5.83 | 4.30 |
|  | *L. griseus* | gray snapper | 89 |  | 14.08 | 0.00 |  | 8.10 | 2.20 |
|  | *Ocyurus chrysurus* | yellowtail snapper | 86 |  | 40.50 | 33.30 |  | 67.62 | 37.00 |
|  | *L. apodus* | schoolmaster | 67 |  | 17.88 | 26.70 |  | 48.05 | 66.30 |
|  | *L. synagris* | lane snapper | 60 |  | 6.32 | 0.00 |  | 16.37 | 4.30 |
|  | *L. mahogoni* | mahogany snapper | 48 |  | 21.05 | 0.00 |  | 31.92 | 45.70 |
| Serranidae | *Mycteroperca bonaci* | black grouper | 148 |  | 0.52 | 0.00 |  | 0.40 | 1.10 |
|  | *Epinephelus striatus* | Nassau grouper | 122 |  | 0.48 | 0.00 |  | 2.28 | 3.30 |
|  | *M. tigris* | tiger grouper | 101 |  | 1.85 | 20.00 |  | 1.20 | 6.50 |
|  | *M. venenosa* | yellowfin grouper | 100 |  | 0.52 | 0.00 |  | 0.00 | 3.30 |
|  | *E. guttatus* | red hind | 76 |  | 0.00 | 0.00 |  | 0.03 | 0.00 |
|  | *E. adscensionis* | rock hind | 61 |  | 10.65 | 33.30 |  | 2.10 | 1.10 |
|  | *Cephalopholis cruentata* | graysby | 43 |  | 58.30 | 53.30 |  | 51.00 | 55.40 |
|  | *C. fulva* | coney | 41 |  | 50.60 | 53.30 |  | 43.40 | 88.00 |
| Sphyraenidae | *Sphyraena barracuda* | barracuda | 200 |  | 16.53 | 46.70 |  | 22.13 | 62.00 |
| ^a^ Excluding the relatively unfished trumpetfish, SF% of fishes larger than 100cm was 2.98 times higher on Navassa Island and 2.43 times higher on Mona Island, compared to Jamaica and Puerto Rico, respectively | | | | | | | | | |
| ^b^ Size data for sharks and jacks are from Caribbean reef shark (*Carcharhinus perezii*) and bar jack (*Caranx ruber*), respectively, which were the most common | | | | | | | | | |
| family representatives | |  |  |  |  |  |  |  |  |
