# Supplementary material for: Fishery-Independent Data Reveal Negative Effect of Human Population Density on Caribbean Predatory Fish Communities
Source: PLoS One. 2009 May 6;4(5):e5333. doi: 10.1371/journal.pone.0005333 (PMC2672166; doi:10.1371/journal.pone.0005333)
Supplement: Table S2 — Regression statistics of the presence of predatory reef fishes across time (1994–2008) by human population density interaction. (0.05 MB DOC) [file pone.0005333.s005.doc]

**On-line supplementary material**

Table S2. Regression statistics^a^ of the presence of predatory reef fishes across a time (1994-2008) by human population density

interaction.

| Family | Taxa | Common name | TL_max_ (cm) | Intercept | SE | Coef | SE | *t-Value* | *p-Value* |
| --- | --- | --- | --- | --- | --- | --- | --- | --- | --- |
| Aulostomidae | *Aulostomus maculatus* | trumpetfish | 100 | 0.6236 | 0.0731 | 0.0000 | 0.0001 | 0.34 | 0.733 |
| Carangidae | *Caranx* spp. | jacks | 69^b^ | 0.8621 | 0.0635 | 0.0001 | 0.0001 | 0.82 | 0.417 |
| Carcharhinidae | *Carcharhinus* spp. | requiem sharks | 300^b^ | 0.0781 | 0.0225 | 0.0000 | 0.0000 | 0.53 | 0.601 |
| Lutjanidae | *Lutjanus cyanopterus* | cubera snapper | 160 | 0.1023 | 0.0491 | 0.0000 | 0.0001 | 0.34 | 0.732 |
|  | *L. jocu* | dog snapper | 128 | 0.2216 | 0.0545 | 0.0001 | 0.0001 | 1.50 | 0.138 |
|  | *L. analis* | mutton snapper | 94 | 0.2472 | 0.0457 | 0.0001 | 0.0001 | 0.80 | 0.428 |
|  | *L. griseus* | gray snapper | 89 | 0.2200 | 0.0321 | 0.0001 | 0.0000 | 1.10 | 0.276 |
|  | *Ocyurus chrysurus* | yellowtail snapper | 86 | 0.8402 | 0.0648 | 0.0000 | 0.0001 | 0.51 | 0.613 |
|  | *L. apodus* | schoolmaster | 67 | 0.6854 | 0.0787 | 0.0001 | 0.0001 | 0.78 | 0.438 |
|  | *L. synagris* | lane snapper | 60 | 0.0635 | 0.0309 | 0.0001 | 0.0000 | 1.54 | 0.129 |
|  | *L. mahogoni* | mahogany snapper | 48 | 0.3779 | 0.0720 | 0.0000 | 0.0001 | 0.45 | 0.657 |
| Serranidae | *Mycteroperca bonaci* | black grouper | 148 | 0.2297 | 0.0529 | 0.0000 | 0.0001 | -0.45 | 0.655 |
|  | *Epinephelus striatus* | Nassau grouper | 122 | 0.5275 | 0.0799 | 0.0001 | 0.0001 | 1.03 | 0.306 |
|  | *M. tigris* | tiger grouper | 101 | 0.3049 | 0.0669 | 0.0000 | 0.0001 | -0.43 | 0.665 |
|  | *M. venenosa* | yellowfin grouper | 100 | 0.0407 | 0.0085 | 0.0000 | 0.0000 | -0.09 | 0.930 |
|  | *E. guttatus* | red hind | 76 | 0.3001 | 0.0621 | 0.0001 | 0.0001 | 1.09 | 0.278 |
|  | *E. adscensionis* | rock hind | 61 | 0.1225 | 0.0238 | 0.0000 | 0.0000 | 0.46 | 0.646 |
|  | *Cephalopholis cruentata* | graysby | 43 | 0.5684 | 0.0771 | 0.0001 | 0.0001 | 0.78 | 0.440 |
|  | *C. fulva* | coney | 41 | 0.5499 | 0.0795 | 0.0001 | 0.0001 | 0.76 | 0.449 |
| Sphyraenidae | *Sphyraena barracuda* | barracuda | 200 | 0.5808 | 0.0670 | 0.0000 | 0.0001 | 0.16 | 0.870 |
| ^a^ Regression coefficient and intercept values computed from untransformed data; test statistics computed from arcsine(x^0.5) transformed data (Zar 1999) | | | | | | | | | |
| ^b^ Size data for sharks and jacks are from Caribbean reef shark (*Carcharhinus perezii*) and bar jack (*Caranx ruber*), respectively, which were the most common family representatives | | | | | | | | | |
