# Supplementary material for: Fishery-Independent Data Reveal Negative Effect of Human Population Density on Caribbean Predatory Fish Communities
Source: PLoS One. 2009 May 6;4(5):e5333. doi: 10.1371/journal.pone.0005333 (PMC2672166; doi:10.1371/journal.pone.0005333)
Supplement: Table S3 — Regression statistics of the presence of predatory reef fishes across time (1994–2008). (0.05 MB DOC) [file pone.0005333.s006.doc]

**On-line supplementary material**

Table S3. Regression statistics^a^ of the presence of predatory reef fishes across time (1994-2008).

| Family | Taxa | Common name | TL_max_ (cm) | Intercept | SE | Coef | SE | *t-Value* | *p-Value*^c^ |
| --- | --- | --- | --- | --- | --- | --- | --- | --- | --- |
| Aulostomidae | *Aulostomus maculatus* | trumpetfish | 100 | 0.7073 | 0.0537 | -0.0468 | 0.0162 | -2.97 | 0.004^*^ |
| Carangidae | *Caranx* spp. | jacks | 69^b^ | 0.7930 | 0.0421 | -0.0183 | 0.0127 | -1.70 | 0.093 |
| Carcharhinidae | *Carcharhinus* spp. | requiem sharks | 300^b^ | 0.0471 | 0.0161 | -0.0024 | 0.0049 | -0.59 | 0.559 |
| Lutjanidae | *Lutjanus cyanopterus* | cubera snapper | 160 | 0.0679 | 0.0325 | -0.0055 | 0.0098 | -0.21 | 0.831 |
|  | *L. jocu* | dog snapper | 128 | 0.1439 | 0.0363 | -0.0147 | 0.0110 | -1.38 | 0.171 |
|  | *L. analis* | mutton snapper | 94 | 0.1871 | 0.0310 | -0.0080 | 0.0093 | -0.78 | 0.437 |
|  | *L. griseus* | gray snapper | 89 | 0.1649 | 0.0221 | -0.0123 | 0.0067 | -1.77 | 0.081 |
|  | *Ocyurus chrysurus* | yellowtail snapper | 86 | 0.7577 | 0.0453 | -0.0153 | 0.0137 | -1.45 | 0.151 |
|  | *L. apodus* | schoolmaster | 67 | 0.5109 | 0.0607 | -0.0036 | 0.0183 | -0.46 | 0.644 |
|  | *L. synagris* | lane snapper | 60 | 0.0530 | 0.0207 | 0.0116 | 0.0063 | 2.14 | 0.036 |
|  | *L. mahogoni* | mahogany snapper | 48 | 0.3958 | 0.0474 | 0.0060 | 0.0143 | 0.29 | 0.776 |
| Serranidae | *Mycteroperca bonaci* | black grouper | 148 | 0.1342 | 0.0430 | -0.0032 | 0.0130 | -0.34 | 0.734 |
|  | *Epinephelus striatus* | Nassau grouper | 122 | 0.3045 | 0.0671 | -0.0200 | 0.0202 | -1.06 | 0.292 |
|  | *M. tigris* | tiger grouper | 101 | 0.2153 | 0.0496 | -0.0151 | 0.0150 | -1.36 | 0.180 |
|  | *M. venenosa* | yellowfin grouper | 100 | 0.0268 | 0.0063 | -0.0015 | 0.0019 | -0.38 | 0.707 |
|  | *E. guttatus* | red hind | 76 | 0.2684 | 0.0407 | -0.0076 | 0.0123 | -0.97 | 0.334 |
|  | *E. adscensionis* | rock hind | 61 | 0.1148 | 0.0154 | -0.0114 | 0.0046 | -2.58 | 0.012 |
|  | *Cephalopholis cruentata* | graysby | 43 | 0.5436 | 0.0500 | 0.0010 | 0.0151 | 0.01 | 0.991 |
|  | *C. fulva* | coney | 41 | 0.5255 | 0.0517 | -0.0083 | 0.0156 | -0.56 | 0.579 |
| Sphyraenidae | *Sphyraena barracuda* | barracuda | 200 | 0.4803 | 0.0491 | -0.0375 | 0.0148 | -2.58 | 0.012 |
| ^a^ Regression coefficient and intercept values computed from untransformed data; test statistics computed from arcsine(x^0.5) transformed data (Zar 1999) | | | | | | | | | |
| ^b^ Size data for sharks and jacks are from Caribbean reef shark (*Carcharhinus perezii*) and bar jack (*Caranx ruber*), respectively, which were the most common family representatives | | | | | | | | | |
| ^C^ Significant test after correction for multiple comparisons using sequential Bonferroni noted (*) | | | | | |  |  |  |  |
