# Supplementary material for: Fishery-Independent Data Reveal Negative Effect of Human Population Density on Caribbean Predatory Fish Communities
Source: PLoS One. 2009 May 6;4(5):e5333. doi: 10.1371/journal.pone.0005333 (PMC2672166; doi:10.1371/journal.pone.0005333)
Supplement: Table S4 — Tests of whether sighting frequency differed between novice and expert surveyors. (0.05 MB DOC) [file pone.0005333.s007.doc]

**On-line supplementary material**

Table S4. Statistics from 40 ANCOVA tests of whether sighting frequency differed between novice and expert surveyors across a gradient of human population density (HPD) and latitude (Lat).

| Family | Taxa | Common name | HPD coef | SE | *t-Value* | *p-Value* | Lat coef | SE | *t-Value* |
| --- | --- | --- | --- | --- | --- | --- | --- | --- | --- |
| Aulostomidae | *Aulostomus maculatus* | trumpetfish | 0.0081 | 0.0224 | 0.362 | 0.718 | 0.1979 | 0.8503 | 0.233 |
| Carangidae | *Caranx* spp. | jacks | 0.0166 | 0.0170 | 0.976 | 0.331 | -0.3693 | 0.6809 | -0.543 |
| Carcharhinidae | *Carcharhinus* spp. | requiem sharks^a^ | 0.0002 | 0.0002 | 0.839 | 0.403 | -0.0022 | 0.0099 | -0.228 |
| Lutjanidae | *Lutjanus cyanopterus* | cubera snapper^a^ | 0.0004 | 0.0003 | 1.374 | 0.172 | -0.0109 | 0.0117 | -0.925 |
|  | *L. jocu* | dog snapper | -0.0032 | 0.0107 | -0.296 | 0.768 | 0.1968 | 0.4373 | 0.450 |
|  | *L. analis* | mutton snapper^a^ | 0.0003 | 0.0002 | 1.248 | 0.214 | -0.0060 | 0.0082 | -0.735 |
|  | *L. griseus* | gray snapper | 0.0122 | 0.0126 | 0.961 | 0.339 | -0.6067 | 0.4998 | -1.214 |
|  | *Ocyurus chrysurus* | yellowtail snapper | 0.0126 | 0.0194 | 0.649 | 0.518 | -0.1231 | 0.8552 | -0.144 |
|  | *L. apodus* | schoolmaster | 0.0380 | 0.0243 | 1.564 | 0.120 | -1.1375 | 1.0217 | -1.113 |
|  | *L. synagris* | lane snapper | 0.0034 | 0.0119 | 0.288 | 0.774 | -0.0613 | 0.4997 | -0.123 |
|  | *L. mahogoni* | mahogany snapper | -0.0001 | 0.0229 | -0.005 | 0.996 | 0.7294 | 0.7449 | 0.979 |
| Serranidae | *Mycteroperca bonaci* | black grouper^a^ | 0.0001 | 0.0002 | 0.342 | 0.733 | -0.0009 | 0.0086 | -0.106 |
|  | *Epinephelus striatus* | Nassau grouper^a^ | -0.0001 | 0.0001 | -1.315 | 0.191 | 0.0037 | 0.0027 | 1.368 |
|  | *M. tigris* | tiger grouper | 0.0240 | 0.0211 | 1.138 | 0.257 | -1.4381 | 0.8309 | -1.731 |
|  | *M. venenosa* | yellowfin grouper^a^ | 0.0003 | 0.0003 | 1.181 | 0.240 | -0.0181 | 0.0118 | -1.534 |
|  | *E. guttatus* | red hind^a^ | 0.0000 | 0.0001 | 0.018 | 0.986 | -0.0027 | 0.0059 | -0.456 |
|  | *E. adscensionis* | rock hind | -0.0017 | 0.0059 | -0.291 | 0.772 | 0.0002 | 0.2404 | 0.001 |
|  | *Cephalopholis cruentata* | graysby^a^ | 0.0000 | 0.0002 | 0.195 | 0.846 | 0.0035 | 0.0092 | 0.380 |
|  | *C. fulva* | coney | 0.0412 | 0.0298 | 1.385 | 0.169 | -0.4250 | 1.1733 | -0.362 |
| Sphyraenidae | *Sphyraena barracuda* | barracuda | 0.0114 | 0.0200 | 0.570 | 0.570 | -0.1873 | 0.8060 | -0.232 |
| ^a^ Regression coefficient values computed from untransformed data; test statistics computed from arcsine(x^0.5) transformed data (Zar 1999) | | | | | | | | | |
| *Note*: P-values were not corrected for multiple comparisons and therefore provide conservative justification that novice and expert sighting frequencies did not differ across the gradient of human population density. | | | | | | | | | |
